# Wireless Sensor for Meat Freshness Assessment Based on Radio Frequency Communication

**DOI:** 10.1021/acssensors.3c01657

**Published:** 2024-02-07

**Authors:** Rafaela S. Andre, Rodrigo Schneider, Guilherme R. DeLima, Lucas Fugikawa-Santos, Daniel S. Correa

**Affiliations:** † Nanotechnology National Laboratory for Agriculture (LNNA), Embrapa Instrumentação, 13560-970 São Carlos, SP, Brazil; ‡ PPGQ, Department of Chemistry, Center for Exact Sciences and Technology, Federal University of Sao Carlos (UFSCar), 13565-905 Sao Carlos, SP, Brazil; § Institute of Biosciences, Letters and Exact Sciences, São Paulo State University – UNESP, 15054-000 São José do Rio Preto, SP, Brazil; ∥ Institute of Geosciences and Exact Sciences, São Paulo State University – UNESP, 13506-900 Rio Claro, SP, Brazil

**Keywords:** wireless communication, radio frequency, sensors, ammonia, meat freshness

## Abstract

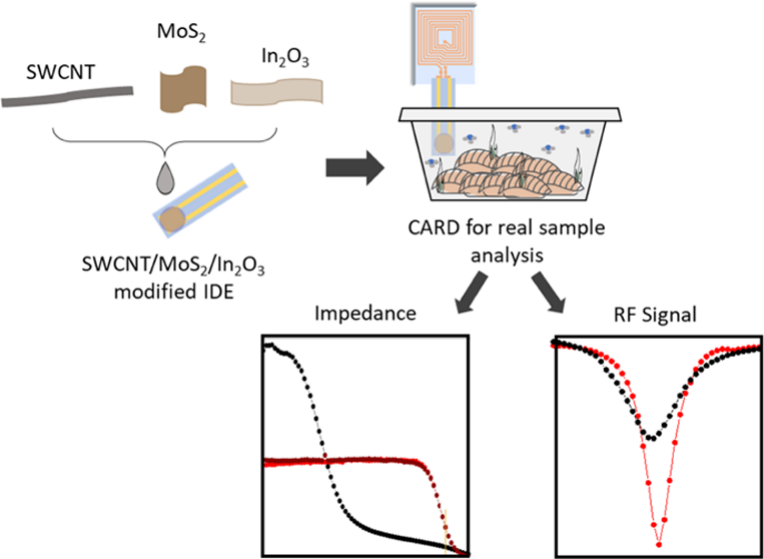

Wireless communication technologies, particularly radio frequency (RF), have been widely explored for wearable electronics with secure and user-friendly information transmission. By exploiting the operational principle of chemically actuated resonant devices (CARDs) and the electrical response observed in chemiresistive materials, we propose a simple and hands-on alternative to design and manufacture RF tags that function as CARDs for wireless sensing of meat freshness. Specifically, the RF antennas were meticulously designed and fabricated by lithography onto a flexible substrate with conductive tape, and the RF signal was characterized in terms of amplitude and peak resonant frequency. Subsequently, a single-walled carbon nanotube (SWCNT)/MoS_2_/In_2_O_3_ chemiresistive composite was incorporated into the RF tag to convey it as CARDs. The RF signal was then utilized to establish a correlation between the sensor’s electrical response and the RF attenuation signal (reflection coefficient) in the presence of volatile amines and seafood (shrimp) samples. The freshness of the seafood samples was systematically assessed throughout the storage time by utilizing the CARDs, thereby underscoring their effective potential for monitoring food quality. Specifically, the developed wireless tags provide cumulative amine exposure data within the food package, demonstrating a gradual decrease in radio frequency signals. This study illustrates the versatility of RF tags integrated with chemiresistors as a promising pathway toward scalable, affordable, and portable wireless chemical sensors.

## 1 Introduction

Integrated sensors with data transmission and wireless communication have gained tremendous importance over the past few years in order to enable new alternatives for real-time monitoring and fast and dynamic response to different analytes.^1−3^ Wireless communication technologies, such as radio frequency (RF), have been widely explored in wearable electronics, providing secure and user-friendly information transmission.^3,4^ RF enables complete integration with sensor devices, eliminating the need for batteries in passive mode and allowing rapid data transmission through RF signals for contactless identification.^5,6^ Although the initial research on RFID dates back to the 1940s, the technology has taken several decades to reach its current state.^7^

Radio-frequency identification (RFID) technology has been applied in various areas, such as electronic toll collection, asset identification, retail item management, access control, and animal and vehicle tracking.^8−10^ RFID readers can also be configured to incorporate additional data, such as environmental parameters.^11^ Another advantage of RFID technology lies in its ability to read and distinguish different tags in short distances, thereby enhancing the reliability of the data collection. An RFID system typically comprises a reader, a tag (or transponder), and an encoding–decoding communication protocol. The reader generates and sends an interrogation signal, which the transponder receives, decodes, and interprets. The tag response is encoded and sent back to the reader, which processes the information. Depending on the type of application, the data transmitted from the transponder activate an actuator or initiate an automated action. It can also be stored or displayed for reading, verification, or monitoring purposes.^10^ Some readers are integrated into more sophisticated devices such as smartphones, microprocessors, and computers, operating through appropriate software, firmware, or mobile applications.^3^

The resonant frequency of an RFID tag is determined by precisely tuning the impedance of a simple LC circuit consisting of a conductive coil (which also serves as the device’s antenna) and a matching capacitor. The tag is fabricated by connecting a microchip for data transceiving through the resonant antenna, with all components coupled to a substrate, which can vary in shape and size.^12^ The digitally controlled microchip stores the data, while the antenna transmits the data. Regarding energy supply, tags can be classified as active and passive. Active tags require an attached battery for operation, resulting in higher reliability, extended operation ranges, and support for more complex circuitry.^13^ On the other hand, passive tags rely on the energy provided by the electromagnetic wave emitted by the reader to operate.^14^ Passive tags offer the advantages of simpler architectures, lightweight design, and long-lasting performance. However, they have a shorter operation range and can support less sophisticated circuitry.^15^ The components attached to the tags can influence the size and shape of the tags, thereby determining the choice of materials, manufacturing techniques and design. These factors are crucial in defining the device quality factor, which directly impacts the performance of the tags.^16^

Recently, there has been a notable increase in the exploration of integration between chemical sensors and cutting-edge wireless technologies like RFID. This convergence has found application in the development of intelligent packaging for monitoring food quality and safety.^3,17,18^ Azzarelli et al. presented for the first time the conversion of RFID tags into chemically actuated resonant devices (CARDs) by incorporating chemical detection species in parallel with the RF tag circuit.^19−21^ The chemical sensing information collected from the environment around the CARD was converted and transmitted by the resonant frequency of the device (f_0_) and its amplitude, also called gain, and given in decibels (dB).^22^ CARDs are ideal for monitoring volatile compounds that are food quality indicators. They do not require direct contact with the sample and enable contactless data transmission between the sensor and the reading device, allowing analysis without food packaging violation.^23,24^

**Scheme 1:**
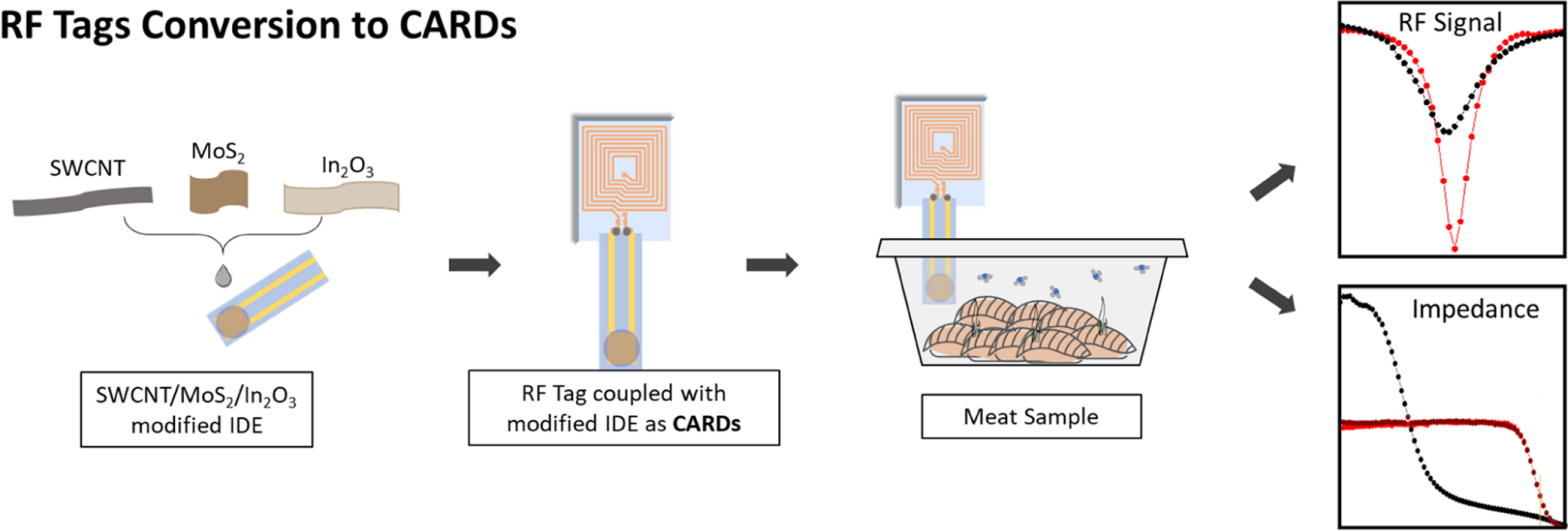
Conversion of the RF Tag into CARDs with SWCNT/MoS_2_/In_2_O_3_ Nanomaterials for Amine Detection over Meat Spoilage by the RF Signal or Electrical Impedance Changes

Food products, like meat, are highly perishable due to their complex and dynamic nature, where each constituent changes rapidly and persistently.^25,26^ The primary process of meat decomposition produces biogenic amines, also known as off-flavors, due to their unpleasant smell, and their concentration levels can be related to the freshness of the meat, with concentrations above 25 ppm indicative of meat unsuitable for consumption.^26^ Chemiresistive materials have been extensively investigated for detecting volatile compounds, owing to their remarkable electrical property variations upon exposure to such compounds.^27−29^ Given the operational principle of CARDs and the observed electrical response in chemiresistive materials,^2^ the combination of these two components emerges as a potential alternative strategy for detecting amines. In this context, here we propose the fabrication of affordable and scalable radio frequency tags meticulously designed and manufactured using optimized materials to operate efficiently within the high-frequency (HF) range (∼13 MHz) to detect different concentrations of amines. The fabricated antenna was characterized as a function of the RF signal amplitude and peak resonant frequency before and after incorporation of the SWCNT/MoS_2_/In_2_O_3_ chemiresistive composite for the CARD conceiving. The composite material was characterized by several techniques, including transmission electron microscopy (TEM), Raman spectroscopy, atomic force microscopy (AFM), and electrical impedance spectroscopy. After the CARD assemblage, the RF signal was pivotal in correlating the sensor’s electrical response with the RF attenuation signal (reflection coefficient). Notably, the efficacy of the CARD was demonstrated through the evaluation of food samples over time of storage, showcasing its potential in monitoring food quality during storage, as schematically illustrated in Scheme 1.

## 2 Results and Discussion

**Figure 1:**
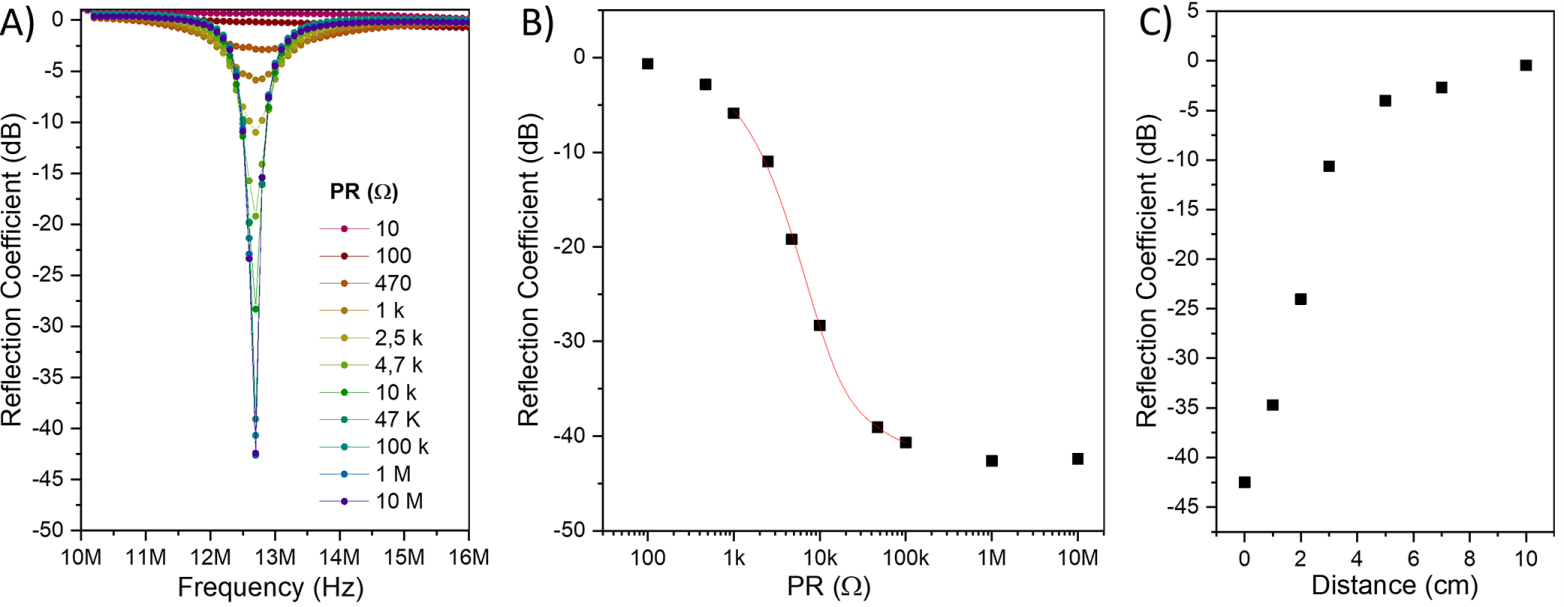
Reflection coefficient for the antenna (A) with different values of parallel resistance (PR (Ω)) as a function of frequency, (B) as a function of parallel resistance value, and (C) as a function of distance between the RF tag and the reader.

The antennas, obtained by photolithography (Figure S1B) onto a flexible PET substrate (Figure S1B(i)), presented a measured inductance of 1.38 μH with the RF signal centered at 12.7 MHz and a maximum reflection coefficient of −43 dB (Figure S1C). The theoretical resonance frequency is 13.56 MHz, while the observed resonance frequency is 0.86 MHz lower. Such a difference in resonant frequency might occur due to the impedance mismatch caused by the SMD capacitor value variation (10% of nominal value) and the parasitic inductive/capacitive reactance from the SMD electrical contact. The RF reflectance response of the tag (a copper loop in the PET substrate with the SMD capacitor) was evaluated according to the variation of an electrical resistance coupled in parallel with the RF tag (Figure 1A,B). Such resistance was combined in parallel with the antenna using commercial resistors with known electrical resistance values. The RF reflection coefficient reaches its maximum value when the parallel resistance is high, equivalent to an open-circuit situation. As the parallel resistance decreases, the reflection coefficient decreases significantly due to substantial energy dissipation from the electromagnetic wave by the parallel resistor.^100^ When the parallel resistance reaches low values, typically below a few hundred Ohms (Ω), the RF signal diminishes to zero. This occurs because the condition becomes similar to short-circuiting the antenna/capacitor circuit, resulting in minimal RF signal transmission (Figure 1B).^30^ Thus, for values between 100 kΩ and 10 MΩ, the reflection coefficient is similar to the antenna without any resistor (−43 dB), indicating that 100 kΩ represents the limit value above which there is no influence of the parallel resistance on the RF signal. For values between 500 Ω and 50 kΩ, the RF signal varies linearly with the resistance value, ranging from −40 to −4 dB.

By identifying the region exhibiting the highest RF signal variation, it was possible to refine the selection of materials capable of offering the desired electrical resistance value within that specific range. The objective was to achieve substantial variation in the RF signal when exposing the CARD to the target analytes. Such narrowed selection aimed to optimize the sensor’s sensitivity and responsiveness, enabling effective detection and monitoring of the desired analytes. The RF signal attenuation was investigated as a function of the distance between the RF tag and the reader antenna (Figure 1C). The reflection coefficient decreases as the RF tag moves away from the antenna. The RF high gain at ∼0 cm, −43 dB, reduces to almost 0 dB at 7 cm, showing a dependence on the distance between the reader and the RF tag. This dependence is expected and is a direct result of the geometric characteristics of the designed tag.^4,16^

**Figure 2:**
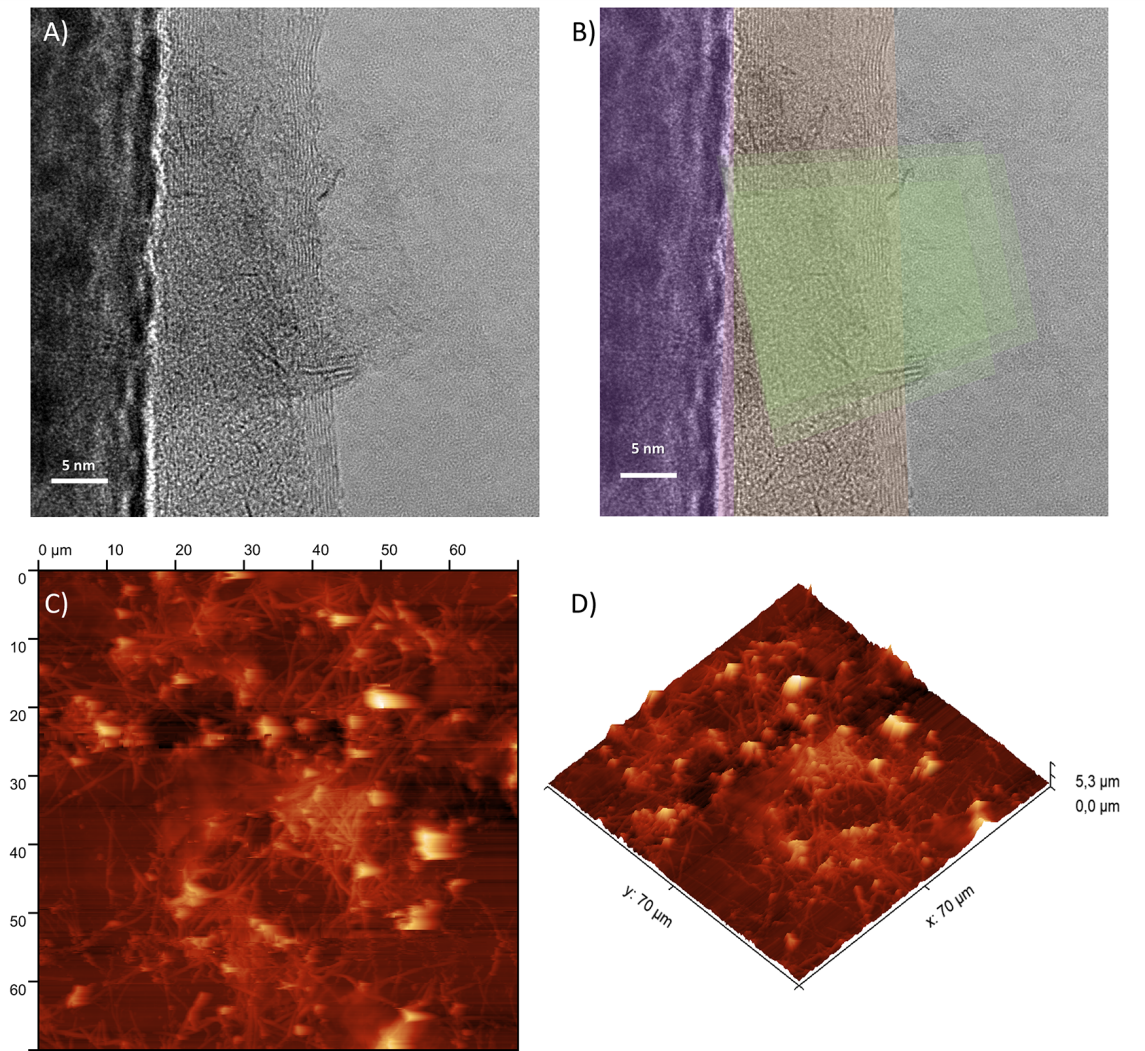
SWCNT/MoS_2_/In_2_O_3_ characterization by (A) the HRTEM image and (B) the artificially colored HRTEM image for In_2_O_3_ nanofibers (purple region), SWCNT (light pink region), and MoS_2_ (highlighted in yellow). (C, D) are AFM images of the materials.

The incorporation of nanomaterials possessing varied geometries (SWCNT-1D, MoS_2_-2D, and In_2_O_3_-1D) confers a distinct advantage to the designed platform once it can foster the creation of a tridimensional interconnected network, thereby enhancing gas percolation properties.^31,32^ TEM characterization of the composite material, presented in Figure 2A,B, showed images of In_2_O_3_ nanofibers as straight and dense structures (purple region) side by side with SWCNT (light pink region), which presented low-density 1D structures. MoS_2_, on the other hand, was identified with 2D sheet morphology (highlighted in yellow) and low density, suggesting a soft packaging with few layers.^33^ Through AFM images (Figure 2C,D), it was possible to characterize the fibrous structure with a 3D porous network, which facilitates gas percolation and adsorption.

Single-walled carbon nanotubes (SWCNT) and molybdenum disulfide (MoS_2_) nanosheets were the materials chosen for investigating the electrical properties and sensitivity to volatile nitrogen compounds. As previously reported elsewhere,^34−36^ these materials were selected due to their unique properties and potential for sensing applications. SWCNT were combined with poly(4-vinylpyridine) (P4VP), and both SWCNT and MoS_2_ were immobilized onto In_2_O_3_ nanofibers (SWCNT/MoS_2_/In_2_O_3_). The electrical properties of the composites were characterized by electrical impedance spectroscopy. The composite SWCNT/MoS_2_/In_2_O_3_ presented an electrical impedance profile that suggests the synergistic effect among the materials with values intermediary to those of pristine materials. The composite material presented impedance values (real components) around 40 kΩ (Figure S2A, green line), which correlates with the beginning of the linear region in RF signal variation (Figure 1B). Pristine SWCNT, SWCNT/In_2_O_3_, and MoS_2_/In_2_O_3_ presented electrical impedance values correspondent to undesired regions of the RF signal as presented in Figure S2 by blue, black (low impedance values), and red line (high impedance values). Based on the electrical characterization results, the composite material SWCNT/MoS_2_/In_2_O_3_ was selected as the chemiresistive material for converting the RF tags into CARDs. In other words, the specific combination of the chosen materials (with their good performance as gas sensors and mostly as ammonia sensors) was able to give proper impedance value, capable of imposing an effect onto the antenna RF signal over gas exposure. Besides, the composites present desirable properties such as enhanced electrical conductivity, sensitivity to target analytes, and good stability. Another important feature was the stability behavior as a function of the relative humidity (RH - %), which is presented in Figure S2B. As can be seen, the tertiary composite showed good stability in terms of electrical impedance properties at 70 and 50% RH, whereas lower RH (30 and 15%) conditions were detrimental to the impedance response. With that in mind, all of the following tests were performed at an RH of 50%.

**Figure 3:**
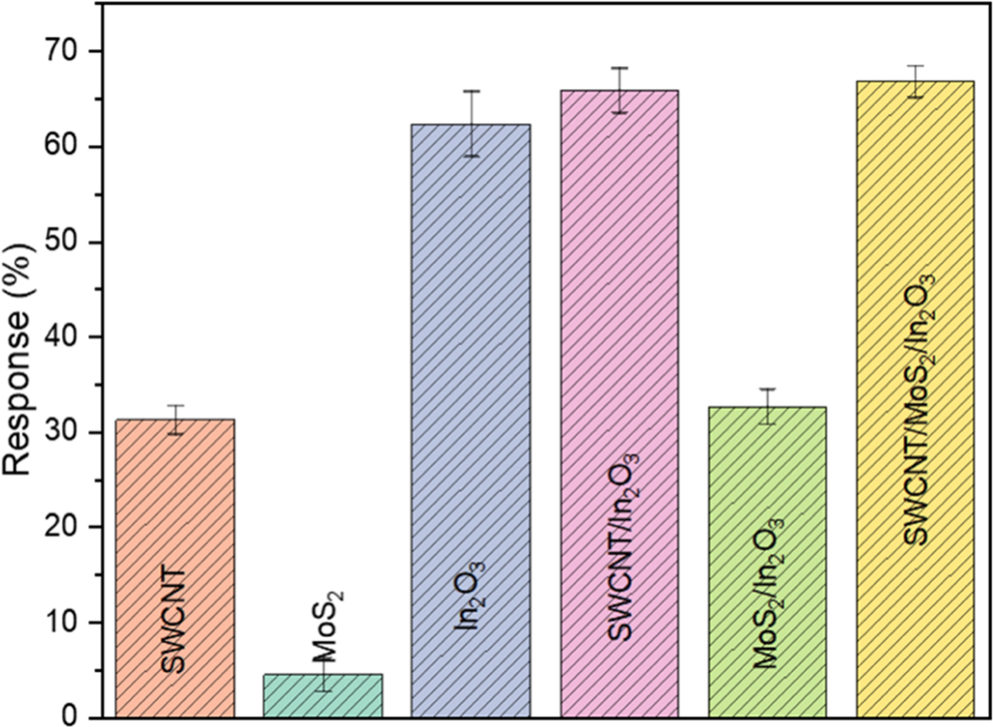
Average device response (*n* ≥ 4) of pristine and composite chemiresistors upon 250 ppm of ammonia.

The sensitivity of pristine materials and composites (Figure 3) upon ammonia exposure was then evaluated by exposing all of them to 250 ppm. In contrast, the electrical impedance was collected and used to calculate the response (%), as described in the Supporting Information. Although In_2_O_3_ and SWCNT/In_2_O_3_ presented significative sensitivity, their initial impedance (*Z*′_0_) values were unsuitable for RF tag conversion into CARDs. Thereby, SWCNT/MoS_2_/In_2_O_3_ was the composite chosen for the RF tag conversion.

**Figure 4:**
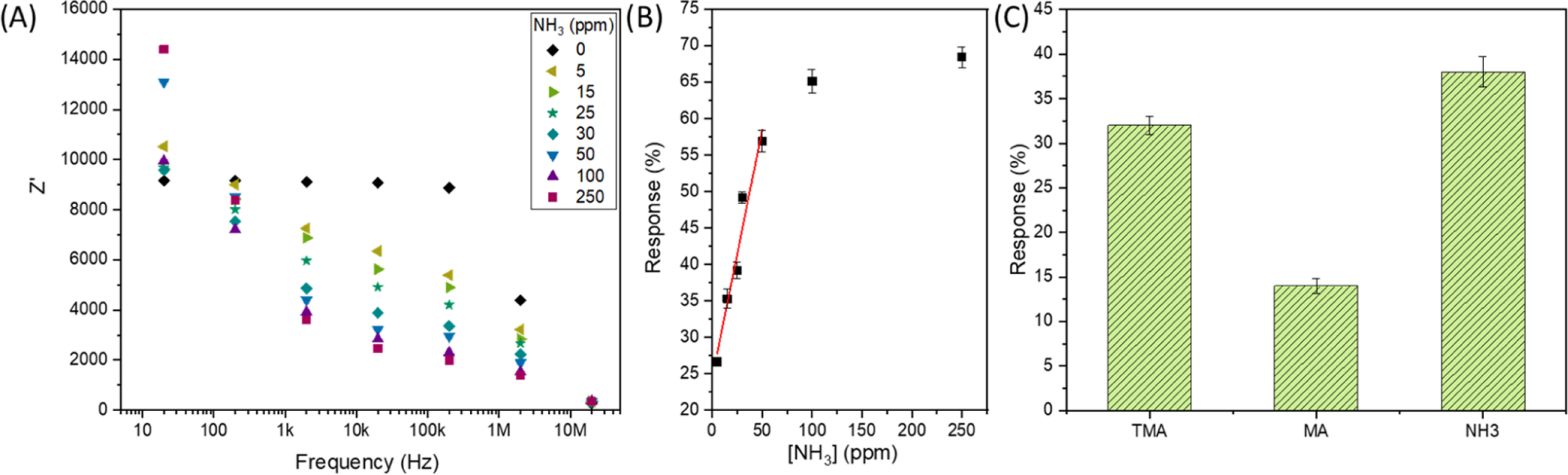
(A) Real component of electrical impedance as a function of frequency when exposed to different concentrations of ammonia, (B) calibration curve, and (C) sensitivity to different amines (TMA, MA, NH_3_).

The sensitivity of the composite was characterized by exposing it to different concentrations of ammonia, while the electrical impedance was measured with an impedance gain/phase analyzer as a function of the frequency (Figure 4A). Ammonia has been chosen because it is the amine with the lowest steric effects and lowest activation energy to interact with the active sites of the sensitive layer when compared with amino groups attached to hydrocarbon chains, such as methylamine or trimethylamine. It is possible to observe the inversion of behavior at a low frequency, 20 Hz, which has been previously observed for other materials and can be attributed to the synergistic effect between materials with resistive behavior (MoS_2_ and In_2_O_3_) and capacitive behavior (SWCNT), resulting in a frequency-dependent response as a function of complex impedance variation.^37^ The calibration curve was carried out for the response regime at 2 MHz (Figure 4B), in which the response was assumed as the variation of the impedance real component values (as described in the Supporting Information, response = Δ*Z*′/*Z*′_0_ × 100%). Thus, the limit of detection (LoD) could be determined as 400 ppb, considering an *S*/*N* ratio equal to 3, where *N* is the standard deviation of the electrical response triplicate and *S* is the angular coefficient of the curve.^38^ The fitting was found to be response = 24.41 + 0.68 [NH_3_] and the respective *R*^2^ = 0.932.

The electrical response behavior under exposure to methylamine (MA), trimethylamine (TMA), and NH_3_ was investigated, as shown in Figure 4C. The proposed composite material demonstrated a response to all three nitrogenous compounds tested, in which electrical response exhibited significant variations in percentage. These findings highlight the potential of the composite material for detecting and monitoring nitrogenous compounds, offering promising prospects for gas-sensing applications. The sensitivity to volatile nitrogenous compounds might be attributed to the synergistic effect among SWCNT/P4VP, MoS_2_, and In_2_O_3_. Specifically, both MoS_2_ and In_2_O_3_ are n-type semiconductors with resistive behavior, and, when in contact with SWCNT, a p-type semiconductor with capacitive behavior, the dominant carriers diffuse toward the interface, leading to the formation of a depletion layer (p–n heterojunction). When exposed to volatile nitrogenous compounds, the amino group interacts with the species adsorbed on the surface of MoS_2_ and In_2_O_3_, shifting the free charge carriers from the interface back to the bulk of the SWCNT, MoS_2_, and In_2_O_3_, resulting in a decrease in the electrical impedance. Besides, the difference in response magnitude for various amines corresponds to the energy for the adsorption processes depending on the alkyl substitution of each volatile.^37^

**Figure 5:**
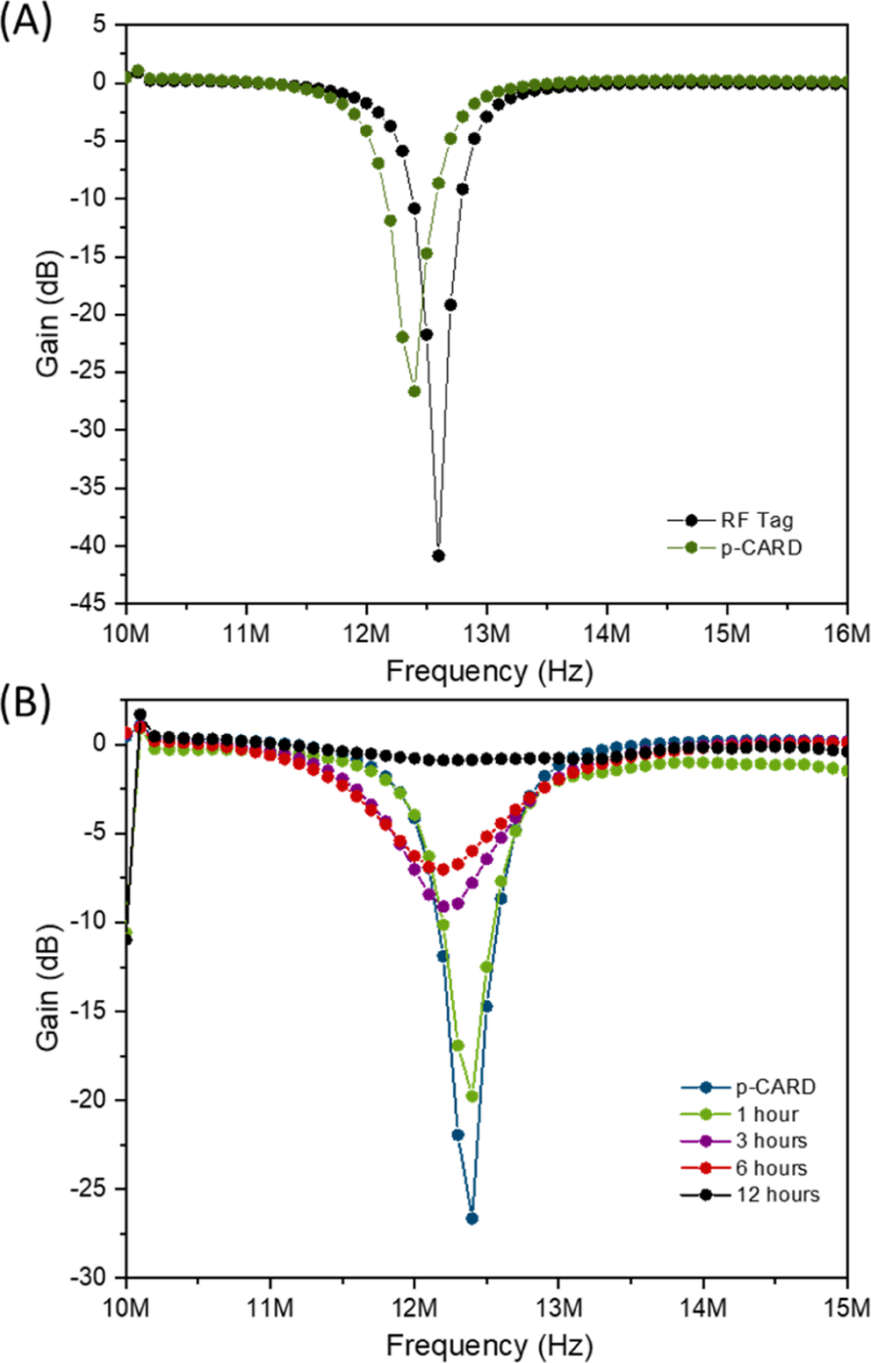
Reflection coefficient of (A) the RF tag and CARD and (B) after specific periods of real sample storage (shrimp).

To evaluate the viability for practical applications as chemically active resonant devices, the RF tags were coupled with modified IDEs as CARDs, as depicted in Scheme 1, and tested in food packages containing food samples to evaluate their freshness through various storage periods. As described in the Supporting Information (Experimental Section), portions of the real sample, seabob shrimp, were placed into plastic containers with the CARD attached to the lid, and the RF signal measurements were collected at time intervals of 1, 3, 6, and 12 h. Figure 5A presents the RF spectra for the region between 10 and 16 MHz. The black trace in Figure 5A represents the signal obtained for the tag made with the copper antennas without functionalization and a reflection coefficient of −43 dB. By the combination of the antenna with the functionalizing material onto IDEs (CARDs), the signal is attenuated to −26 dB, and a shift from 12.7 to 12.4 MHz is observed. This shift might be attributed to parasitic inductive/capacitive reactance from the IDE electrical contact and from the addition of the IDE itself to the original design of the antenna. When placed inside the containers with real samples, the CARD RF spectra showed attenuations of −19, −8, −6, and −1 dB for 1, 3, 6, and 12 h, respectively, corresponding to the shrimp storage time (Figure 5B). According to regulations and good practices,^39^ fresh shrimp are considered fresh within the first 60 min outside of refrigeration and are indicated for consumption after a maximum of 3 h. Therefore, RF signal attenuation for values up to −19 dB can be considered indicative of a fresh product; values between −19 and −8 dB are indicative of a product suitable for consumption but not fresh; and values between −8 and 0 dB are indicative of a product unsuitable for consumption.

## 3 Conclusions

The design and fabrication of passive RF antennas tailored for meat freshness assessment have been successfully achieved by using a combination of SWCNT/MoS_2_/In_2_O_3_, enabling the creation of chemically actuated resonant devices. Electrical characterization has confirmed the synergistic effect among the SWCNT/MoS_2_/In_2_O_3_ constituents. The CARD modification has enabled the modulation of the RF signal, responding to volatile amines associated with meat freshness, by inducing electrical impedance (*Z*′) changes. The specifically designed CARD has demonstrated a robust response to volatile compounds, such as methylamine (MA), trimethylamine (TMA), and ammonia (NH_3_). Validation through real food analysis, employing shrimp meat as a model, has further confirmed the effectiveness of CARD design and the selection of nanomaterials. The dynamic response observed in real food assessment has revealed the CARD’s ability to monitor sample quality by measuring RF attenuation in response to the increment of analyte concentration, reaching values as low as −1 dB. These results underscore the CARD’s potential as a sensible platform for monitoring NH_3_ and other volatile amine compounds related to meat freshness. In summary, integrating SWCNT/MoS_2_/In_2_O_3_ in passive RF antennas has paved the way for developing CARDs, presenting a promising solution for real-time wireless monitoring of meat freshness and the presence of relevant volatile compounds.

## Supporting Information Available

Instrumentation and materials details; tag design and fabrication; sensing materials preparations and synthesis; CARD fabrication, optimization, and characterization; and real sample analysis description and additional photographs of meat package (PDF)
