# Supplementary material for: Wireless Sensor for Meat Freshness Assessment Based on Radio Frequency Communication
Source: ACS Sens. 2024 Feb 7;9(2):631–7. doi: 10.1021/acssensors.3c01657 (PMC11264315; doi:10.1021/acssensors.3c01657)
Supplement: Supplementary file 1 — se3c01657_si_001.pdf [file se3c01657_si_001.pdf]

Supplementary Information

Wireless sensor for meat freshness assessment based on radio-frequency communication

Rafaela S. Andre[1,*], Rodrigo Schneider[1,2], Guilherme R. Lima[3], Lucas Fugikawa-Santos[3,4,*] and Daniel S. Correa[1,2,*]

[1] Nanotechnology National Laboratory for Agriculture (LNNA), Embrapa Instrumentação, 13560-970 São Carlos, SP, Brazil

[2] PPGQ, Department of Chemistry, Center for Exact Sciences and Technology, Federal University of Sao Carlos (UFSCar), 13565-905, Sao Carlos, SP, Brazil

[3] Institute of Biosciences, Letters and Exact Sciences, São Paulo State University - UNESP, 15054-000, São José do Rio Preto, SP, Brazil

[4] Institute of Geosciences and Exact Sciences, São Paulo State University - UNESP, 13506-900 Rio Claro, SP, Brazil

Corresponding authors:
rafaela.s.a@outlook.com
lucas.fugikawa@unesp.br
daniel.correa@embrapa.br

Table of Contents

1. Detailed Experimental Methods 2
1.1. Geral Materials 2
1.2. Geometric Parameters and Tag Design 2
1.3. Tag Fabrication 2
1.4. CARDs Fabrication 3
1.4.1. Sensing Materials Preparation 3
1.4.2. RF tags conversion into CARDs 3
1.5. Characterization 3
1.6. Sensing Measurements 4
2. Supplementary Figures 5
3. References 6

1. Detailed Experimental Methods

1.1. Geral Materials

Single-walled carbon nanotubes (SWCNTs) [(6,5) chirality, ≥ 93% carbon as SWCNT], dimethylformamide (DMF), Indium (III) Chloride ($InCl_3$), poly(vinylpyrrolidone) (PVP, Mw = 1,300,000 g.mol^{-1}), poly(4 vinylpyridine) (PSS, Mw = 75,000 g·mol^{-1}) and negative photoresist I were all purchased from Sigma-Aldrich and used as received. HCl, NaOH and H_2O_2 were purchased from Synth, Brazil. All chemical reagents were analytical grade and used without any further purification.

1.2. Geometric Parameters and Tag Design

To fabricate tags operating in the intended frequency (13.56 MHz), the geometric parameters of the antenna were calculated considering the nominal capacitance of a commercially available SMD capacitor (100 pF), the required inductance (1.38 μH) to achieve the resonant frequency and the coil fulfill ratio assuming an external diameter of square coil (d_{out} = Lx = Ly) of 23 mm. The metal track width (w = 0.5 mm) and the distance of the gap between turns (G = 0.3 mm), shown in Figure S1A, were selected according with the limitations of the fabrication method.[1]

1.3. Tag Fabrication

The tags were fabricated onto flexible substrates (PET), using copper tape patterned by photolithography technique to produce the coil antenna design, represented in scheme S1. The PET substrates were subjected to a cleaning process in an ultrasonic bath containing isopropanol, followed by rinsing in acetone. Subsequently, the substrates were dried using synthetic dry air. After the cleaning and drying process, the copper adhesive tape (3M Electronic Specialty, US) was applied onto the substrate. The copper surface was polished with a steel wool pad to remove any visible oxidized spots. The shadow mask containing the designed tag pattern was imprinted with UV light irradiation for 4 minutes onto the copper tape that was covered with photosensitive ink. The tag pattern was reveled using sodium carbonate solution (10 g.mL^{-1}) (Neon, Brasil) and the excess of copper was removed with an aqueous solution of hydrogen peroxide (15 % w/w) (Synth, Brazil) and hydrochloric acid (10 % w/w) (Synth, Brasil). The SMD capacitor (0603-100pf, 50v) (Vishay, US) was welded onto the tag contact pads as shown in Scheme S1.

1.4. CARDs Fabrication

1.4.1. Sensing Materials Preparation

Single walled carbon nanotubes (SWCNT) functionalized with poly (4 vinyl pyridine) was suspended (0.5 mg mL^{-1}) in DMF and subjected to dispersion in ultrasonic iced bath for 1 hour. P4VP was add to SWCNT to improve the sensitive towards volatile nitrogenated compounds.[2] Pristine molybdenum disulfide (MoS_2) previously obtained[3] was suspended (0.5 mg mL^{-1}) in DMF and subjected to dispersion in ultrasonic bath for 15 minutes. Composite materials (MoS_2/In_2O_3, SWCNT/MoS_2/In_2O_3 e SWCNT/In_2O_3) were prepared by combining the pristine solutions in 1:1 proportion and dispersed in ultrasonic bath for 10 minutes.

1.4.2. RF tags conversion into CARDs

The conversion of the RF tags into CARDs was carried out by the immobilization of the functional nanomaterials onto the tags. Specifically, SWCNT/MoS_2/In_2O_3 was chosen as a sensitive composite for the CARD conversion (Scheme 1). The RF tag was designed with additional pads for adding the material in an interdigitated electrode by drop-casting and welding them to the tag to configure an RC equivalent circuit in parallel with the capacitor and the metallic coil (Fig. S1A-Bi).

1.5. Characterization

1.5.1. Radio-Frequency Signal

The RF tags were tested, and the radio-frequency signal was measured from 10 to 18 MHz using a copper reading antenna connected to a lock-in amplifier (model SR844, Stanford Research Systems) by a non-commercial RF reflectance bridge.[4] The radio-frequency signal was measured before and after converting the antennas into CARDs, before and after exposure to the analytes of interest, and was also evaluated over the storage time of the real samples.

1.5.2. Physico-Chemical Characterization of Chemoresistive Materials

Electrical properties of the pristine and composite materials were characterized by AC electrical impedance in the 2.0 Hz – 2.0 MHz frequency range using an impedance gain/phase analyzer (Solartron 1260).

Morphological and compositional analyses were performed by transmission electron microscopy (TEM) using a FEI Tecnai G2F20 microscope operated with an accelerating voltage of 200 kV. Raman spectroscopy was carried using a LabRAM HR Evolution confocal micro-Raman system and atomic force microscopy (Nanosurf EasyScan) was performed to characterize the arrangement between the pristine and the composite phases.

1.6. Sensing Measurements

Gas detection measurements were performed at room temperature (25 ± 2 °C) using a glass chamber with a Teflon base and lid, as described in a previous work.[5,6] The volatile amines selected for the experiment are Methylamine (MA), Trimethylamine (TMA) and Ammonia (NH_3). The sensitivity and selectivity for different volatile amines and ammonia were studied by exposing the sensor to each volatile analytes at different concentrations while AC electrical impedance was measured as a function of frequency. The response was calculated as Response = $\Delta Z'/Z'_0 \times 100$ (%), being $\Delta Z' = Z'_g - Z'_0$ where Z'_g is the electrical impedance when exposed to the gas, Z'_0 is the electrical impedance in air. The RF reflectance ratio of the antennas was measured using the RF bridge setup when the CARDs were exposed to the analyte and compared to the measurements carried out in the absence of the analyte (carrier gas only).

Real sample analysis was conducted in a plastic container with a capacity of 95 mL in which 9.5 g of the meat sample was packed. The RF tag connected to the electrode containing the sensor material was attached to the lid of the plastic container for in situ measurements while the sample was stored, which generated gases during sample decomposition. The meat used for this study was small seabob shrimp (purchased in a local market), peeled and thawed immediately before the beginning of the experiment. The RF signal analysis experiment was carried out in triplicate. After closing the lids, the containers were sealed with plastic film and the CARDs were interrogated individually over time.

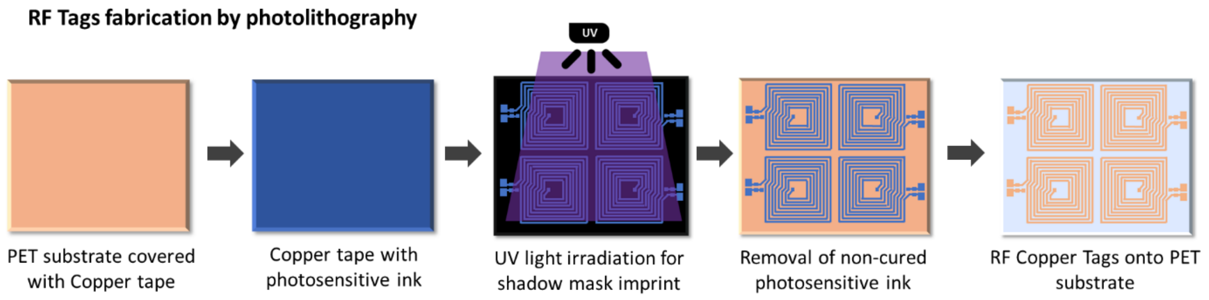

Scheme S1: Schematic representation of RF Tag fabrication process through photolithography. B) Conversion of RF Tag into CARDs with selected nanomaterials for amine detection over meat spoilage by RF signal or electrical impedance changes.

2. Supplementary Figures

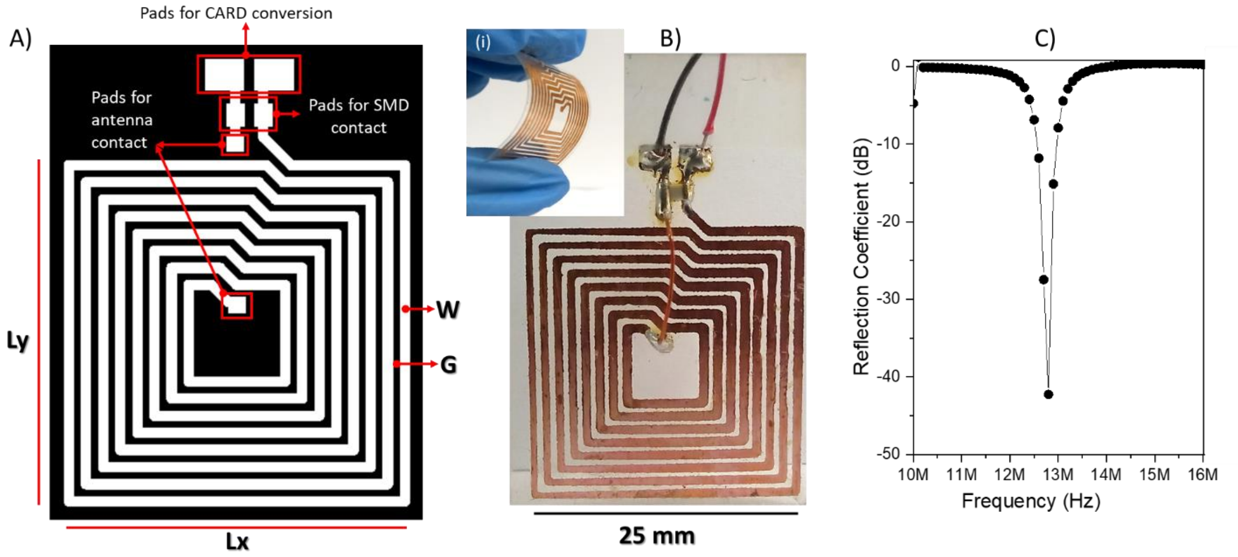

Figure S1: A) Shadow mask and its parameters for FR tag fabrication. B) Digital image of fabricated antennas to be applied as RFID tags and C) RF signal in 12.7 MHz.

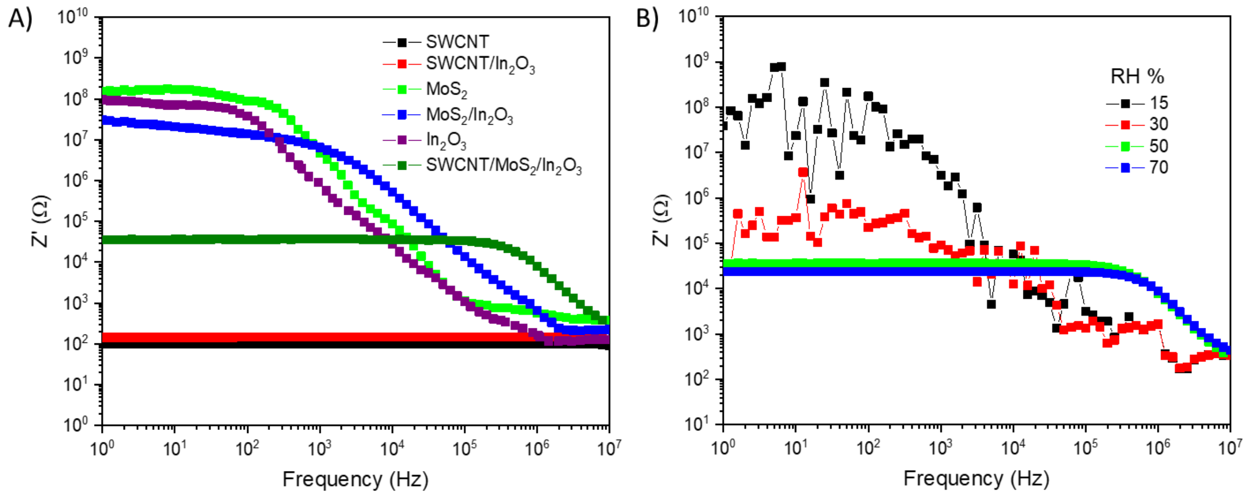

Figure S2: a) Impedance (real component) as a function of frequency for different materials. B) Impedance (real component) of SWCNT/MoS_2/In_2O_3 as function of frequency for different relative humidity levels (RH %).

3. References

(1) Wheeler, H. A. Inductance Formulas for Circular and Square Coils. *Proceedings of the IEEE* **1982**, *70* (12), 1449–1450. https://doi.org/10.1109/PROC.1982.12504.

(2) Tjandra, A. D.; Pham, A.-H.; Chandrawati, R. Polydiacetylene-Based Sensors To Detect Volatile Organic Compounds. *Chemistry of Materials* **2022**, *34* (7), 2853–2876. https://doi.org/10.1021/acs.chemmater.1c04318.

(3) Schneider, R.; Tandel, A. M.; Deng, E.; Correa, D. S.; Lin, H. Scalable Synthesis of Ultrathin MoS2 Membranes for Dye Desalination. *Journal of Membrane Science Letters* **2023**, *3* (2). https://doi.org/10.1016/j.memlet.2023.100058.

(4) De Lima, G. R.; Gozzi, G.; Fugikawa-Santos, L. Lock-in Amplifier as Alternative Instrument for Reading RFID Tags in Sensing Application. 4697846.

(5) Andre, R. S.; Mercante, L. A.; Facure, M. H. M.; Mattoso, L. H. C.; Correa, D. S. Enhanced and Selective Ammonia Detection Using In 2 O 3 /Reduced Graphene Oxide Hybrid Nanofibers. *Appl Surf Sci* **2019**, *473* (August 2018), 133–140. https://doi.org/10.1016/j.apsusc.2018.12.101.

(6) Andre, R. S.; Facure, M. H. M.; Mercante, L. A.; Correa, D. S. Electronic Nose Based on Hybrid Free-Standing Nanofibrous Mats for Meat Spoilage Monitoring. *Sens Actuators B Chem* **2022**, *353*, 131114. https://doi.org/10.1016/j.snb.2021.131114.
